# Longitudinal study on metabolic abnormalities and diabetes risk in normal-weight japanese adults

**DOI:** 10.3389/fendo.2024.1445934

**Published:** 2024-08-29

**Authors:** Cheng Huang, Zhichao Gao, Yuhang Zhang, Guofeng Li

**Affiliations:** ^1^ Department of Colorectal Surgery, First People’s Hospital of Xiaoshan District, Hangzhou, Zhejiang, China; ^2^ Department of Neurosurgery, First People’s Hospital of Xiaoshan District, Hangzhou, Zhejiang, China; ^3^ Department of Orthopedics, First People’s Hospital of Xiaoshan District, Hangzhou, Zhejiang, China

**Keywords:** metabolically unhealthy normal-weight (MUNW), metabolic syndrome (MetS), Japanese adults, diabetes, longitudinal study

## Abstract

**Background:**

Diabetes is a significant global health concern, with a growing prevalence in Japan. Individuals with normal body mass index who are metabolically unhealthy exhibit an elevated risk of diabetes onset. Investigating the relationship between Metabolically Unhealthy Normal-weight (MUNW) status and the risk of diabetes in non-diabetic individual is vital for implementing early preventive strategies.

**Methods:**

Using data from the NAGALA cohort, This study categorized 8,194 Japanese adults based on the score of metabolic syndrome (MetS) components they possessed. Cox proportional hazards regression models and multivariate logistic regression were used to assess the relationship between MUNW and the risk of developing diabetes, with analyses stratified by age and sex.

**Results:**

During an average follow-up of 7.19 years, 123 participants (1.5%) were diagnosed with diabetes. Among the participants, 766 (9.35%) were classified as MUNW, and 157 (1.92%) as having MetS. Compared to those with no MetS components, individuals with 1, 2, and ≥3 MetS components had progressively higher adjusted hazard ratios (HRs) for developing diabetes, at 4.56, 8.79, and 25.16, respectively. Further stratified analysis revealed that men aged ≤40 years had the highest risk of developing diabetes. For men, the adjusted HRs for having 1, 2, and ≥3 MetS components were 5.61, 7.80, and 28.59, respectively, and for participants aged ≤40 years, the HRs were 12.31, 25.57, and 129.82, respectively.

**Conclusion:**

The prevalence of MUNW in non-diabetic individuals in Japan is 9.35%. The score of MetS components was positively correlated with the risk of diabetes. Early intervention and lifestyle modifications are crucial, especially for MUNW individuals and notably young Japanese men aged ≤40 years, for the prevention and management of diabetes.

## Introduction

Diabetes is a serious global health concern of the 21st century and will affect over 570.9 million people worldwide by 2025 (1). It is associated with severe complications like retinopathy, nephropathy, and cardiovascular diseases, leading to significant impacts on health, quality of life, and healthcare expenses (2, 3). East Asia is currently facing a rapidly growing diabetes epidemic that contributes to over 25% of the global diabetes population (4).The global epidemic of obesity and high BMI largely explains the ongoing dramatic increase in the incidence and prevalence of diabetes (5, 6). However, individuals with a normal BMI also can develop diabetes, which is identified as the “metabolically unhealthy normal-weight” (MUNW) phenotype, or “metabolically obese normal weight” (7, 8). Diabetes prevalence is substantial in normal-weight populations, particularly in some Asian countries (13.6-23.5%) (9). At the same BMI, East Asians have 3-5% more body fat than Whites and are more likely to experience adverse metabolic outcomes (10). In Japan, nearly 40% of new-onset diabetes cases occurred in individuals with normal BMI (<22 kg/m²) from 2006-2016 (11). Compared to metabolically healthy overweight individuals, this group is characterized by metabolic abnormalities such as hyperinsulinemia and insulin resistance, resulting in a higher risk of diabetes (12). Recognizing the significance of early intervention, it becomes imperative to elucidate the relationship between the MUNW phenotype and the risk of diabetes in normal weight individuals. Understanding this correlation is crucial for early detection and prevention strategies, as it can help identify individuals who are at a higher risk. In this study, a comprehensive analysis was conducted to explore the relationship between Metabolic Syndrome (MetS) component scores and diabetes risk, as well as to assess the prevalence of diabetes among MUNW individuals in Japan.

## Methods

### Study design and data acquisition

Data were retrieved from the NAGALA cohort to assess the prevalence of MUNW in Japan and to explore the relationship between the score of MetS components and the risk of diabetes among metabolically abnormal individuals with normal weight. The NAGALA cohort data were made publicly available on the open-access database Dryad for general use by Okamura et al (13). According to the terms of the Dryad service, all data that are publicly available in their database can be utilized for secondary analysis and are exempt from ethical review. In the article titled “Ectopic fat obesity presents the greatest risk for incident type 2 diabetes: a population-based longitudinal study”, Okamura et al. provide a detailed description of the study design and introduce the procedures of the NAGALA cohort study (14). The NAGALA cohort represents an extensive longitudinal investigation aimed at evaluating the effectiveness of utilizing the non-high-density lipoprotein to high-density lipoprotein cholesterol ratio (NHHR) as a predictive tool in diabetes prevention strategies. This study delves into the potential of NHHR as a robust indicator for predicting diabetes onset, aiming to provide healthcare professionals with a more refined approach to identifying individuals at risk.The original NAGALA study included participants ranging from 18 to 79 years old, encompassing a total of 15,464 individuals. Among these, 373 were identified with new-onset diabetes between the years 2004 and 2015. Its primary focus is to deepen the understanding of chronic diseases such as fatty liver disease and diabetes, and their impact on public health. Through long-term tracking and observation of participants, the NAGALA cohort aims to reveal the evolution of patient health status, disease progression, and related factors.

### Study population

**Figure 1:**
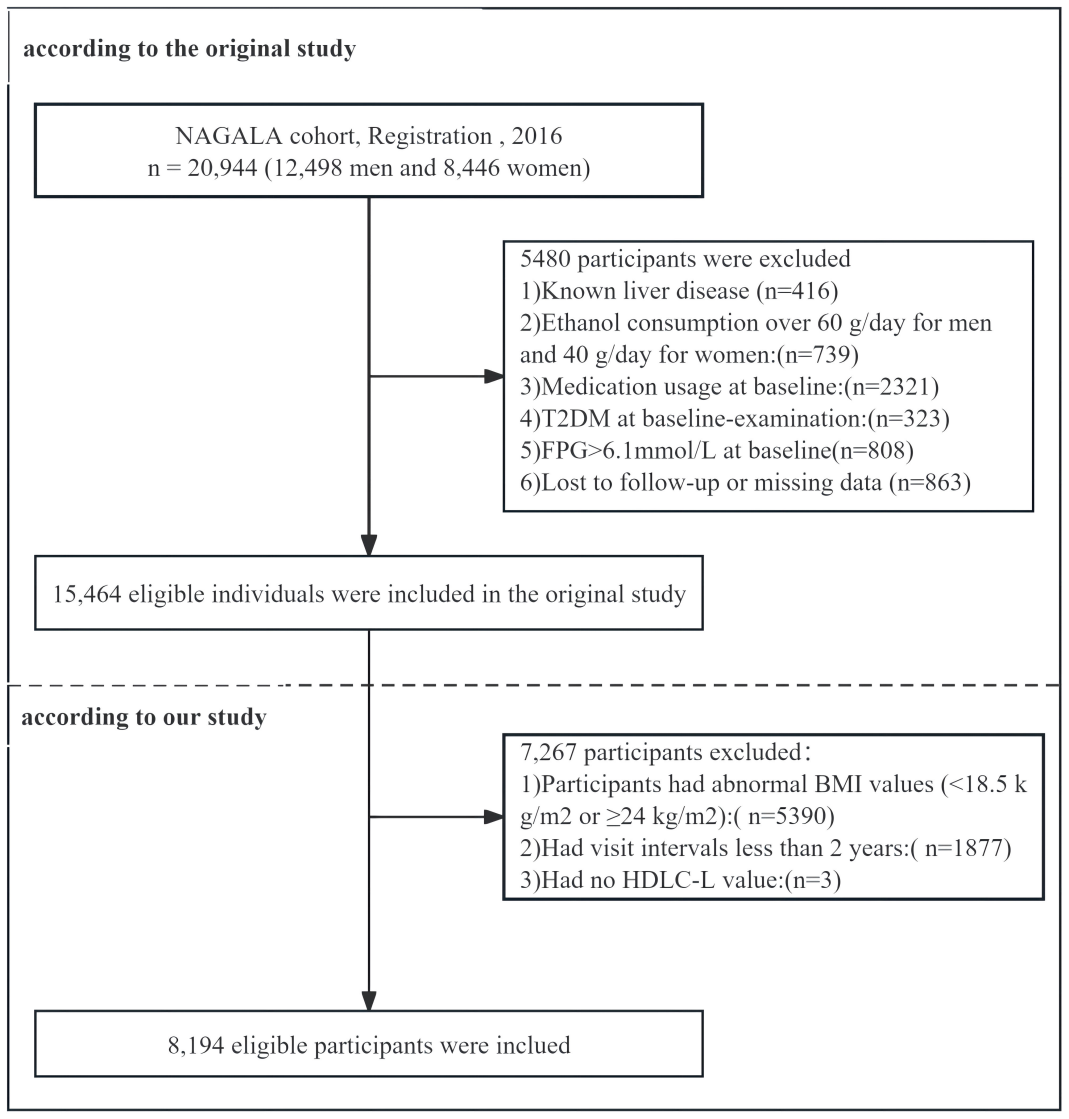
Flowchart of the participant in the study.

In the original study, a total of 5,480 participants were excluded based on specific criteria. The exclusions included those with missing covariate data (863 individuals, comprising 504 males and 359 females), known liver disease (416 individuals, including 278 males and 138 females), and heavy drinking habits, defined as alcohol intake exceeding 40 grams per day for females and 60 grams per day for males (739 participants, including 635 males and 104 females). Furthermore, participants who reported drug use during the baseline examination (2,321 individuals, including 1,709 males and 612 females), those diagnosed with type 2 diabetes at baseline (323 individuals, including 265 males and 58 females), and those with fasting blood glucose levels above 6.1 mmol/L at baseline screening (808 individuals, including 677 males and 131 females) were also excluded. In the final analysis, 15,464 participants were included, of which 8,430 were males and 7,034 were females. Subsequently, this study further excluded 5,390 participants with a BMI <18.5 kg/m² or ≥24 kg/m², 1,877 participants who had a follow-up interval of less than two years, and an additional 3 participants due to missing data on high-density lipoprotein cholesterol (HDL-C). After full exclusion, 8,194 participants (4,268 males and 3,926 females) were included in this study (**Figure 1**).

### Diagnostic criteria for diabetes

The diagnosis of diabetes primarily relied on two criteria: first, an elevated fasting plasma glucose (FPG) level of at least 126 mg/dL (7.0 mmol/L), and second, a glycated hemoglobin (HbA1c) concentration of no less than 6.5%. Furthermore, during the follow-up period, participants who reported having diabetes were also considered to have the condition. participants were considered as censored either at the date of their diabetes diagnosis or upon the completion of the follow-up period, depending on which event occurred first.

### The definition of MUNW and MetS

The metabolic status of participants, including factors such as abdominal obesity, HDL-C levels, triglyceride levels, blood pressure, and fasting glucose levels, was defined according to the guidelines established by the National Cholesterol Education Program Adult Treatment Panel III (NCEP ATP III). All participants in our study had a normal body mass index (BMI); therefore, the waist circumference criterion was not utilized. In this study, participants were diagnosed with MetS if they met three or more of the following criteria: 1) Systolic blood pressure (SBP) exceeding 130 mmHg, diastolic blood pressure (DBP) exceeding 85 mmHg, or the participant receiving hypertension therapy; 2) HDL-C concentrations less than 40 mg/dL (1.04 mmol/L) for males and less than 50 mg/dL (1.30 mmol/L) for females; 3) Serum triglyceride concentrations reaching or surpassing 150 mg/dL (1.7 mmol/L); and 4) FPG concentrations at or above 100.8 mg/dL (5.6 mmol/L), or the participant having a previous diagnosis of diabetes. Each met criterion is assigned a score of “1”. Accordingly, this study divides participants into four groups based on the cumulative score of MetS components: 0, 1, 2, and ≥3. MUNW is characterized by normal-weight individuals (with a BMI between 18.5-24 kg/m²) meeting two or more of the same criteria for MetS.

### Statistical analyses

In this study, data analysis was conducted using EmpowerStats software (X&Y Solutions, Inc, Boston, MA). Continuous variables that exhibited a normal distribution were described using the mean and standard deviation. For continuous variables skewed from normality, the median and interquartile range (IQR) were used to represent the data. Categorical variables are represented using proportions or percentages. In comparing continuous variables across different groups, the analysis of variance (ANOVA) is employed if the data are normally distributed and have homogeneous variances. Conversely, the Kruskal-Wallis test is utilized when the data do not follow a normal distribution or when variances are heterogeneous. For categorical variables, comparisons are made using the chi-square test. Cox proportional hazard model was used to assess the relationship between MetS components score and diabetes incidence. Logistic regression was used to evaluate the association between MUNW status and risk factors, reporting odds ratios (95% CI). Stratification analyses were performed based on age groups (≤40, 40-50, ≥50 years) and gender (male, female), with interaction testing between subgroups. A two-tailed test was conducted with a significance threshold set at p < 0.05.

## Results

### Baseline characteristics of participants

In this study, the research cohort consisted of 8,194 non-diabetic individuals of normal weight, comprising 4,268 males and 3,926 females, with a median age of 43.64 years at baseline (**Table 1**). During an average follow-up of 7.19 years, 1.5% of the participants (123 individuals) were diagnosed with diabetes. The distribution of MetS components was as follows: 5,273 participants (64.35%) had no components; 2,155 (26.30%) had 1 component; 609 (7.43%) had 2 components; and 157 (1.92%) had at least three components. Additionally, 766 individuals (9.35%) were classified as having a MUNW, while 157 (1.92%) met the criteria for MetS. Incident diabetes diagnoses were observed in 22 participants (0.42%) having no MetS components, 49 (2.27%) with one component, 28 (4.60%) with two components, and 24 (15.29%) with at least three components. The mean ages for these respective groups were 42.51 ± 8.27 years, 45.07 ± 9.09 years, 47.30 ± 8.96 years, and 47.96 ± 8.31 years. As the score of MetS components increased, the participants exhibited a consistent rise in BMI, SBP, DBP, liver enzymes (ALT, AST, GGT), total cholesterol (TC), triglycerides (TG) and fasting plasma glucose (FPG), along with a steady decrease in HDL-C. Significant differences were observed in demographic data (age, BMI, smoking, and drinking status) across varying score of MetS components. Univariate logistic regression analysis revealed that the risk of MUNW was significantly associated with both BMI and age, with odds ratios (ORs) of 1.66 (1.57, 1.76) and 1.05 (1.05, 1.06), respectively. Notably, the risk of diabetes was significantly higher in men compared to women, with an OR of 3.26 (2.74, 3.87). Former smokers exhibited a markedly higher risk of diabetes compared to never smokers, with an OR of 2.26 (1.87, 2.72). Current smokers also had a higher risk compared to never smokers, with an OR of 2.12 (1.77, 2.53). The risk of diabetes was also significantly elevated among light, moderate, and heavy drinkers compared to never drinkers, with ORs of 1.29 (1.03, 1.61), 1.68 (1.32, 2.13), and 2.04 (1.45, 2.87), respectively.

**Table 1:** Characteristics of all participants.

	Number of components				
	0	1	2	≥3	P value
Participants, n (%)	5273 (64.35%)	2155 (26.30%)	609 (7.43%)	157 (1.92%)	
Sex (M/F)	2404/2869	1280/875	451/158	133/24	<0.001
Age, years	42.51 ± 8.27	45.07 ± 9.09	47.30 ± 8.96	47.96 ± 8.31	<0.001
≤40	2458 (46.61%)	783 (36.33%)	168 (27.59%)	32 (20.38%)	
40-49	1758 (33.34%)	679 (31.51%)	181 (29.72%)	58 (36.94%)	
≥50	1057 (20.05%)	693 (32.16%)	260 (42.69%)	67 (42.68%)	
BMI (kg/m^2^)	21.03 ± 1.47	21.57 ± 1.45	22.09 ± 1.34	22.75 ± 1.08	<0.001
ALT (IU/L)	16.73 ± 7.79	19.22 ± 20.63	22.02 ± 11.11	26.68 ± 16.08	<0.001
AST (IU/L)	17.32 ± 5.92	18.12 ± 13.86	18.84 ± 5.83	20.82 ± 7.74	<0.001
GGT (IU/L)	16.48 ± 13.32	20.22 ± 18.10	25.15 ± 19.01	33.48 ± 28.02	<0.001
HDL-C (mmol/L)	1.59 ± 0.34	1.34 ± 0.37	1.19 ± 0.34	0.98 ± 0.20	<0.001
TC (mmol/L)	5.03 ± 0.81	5.07 ± 0.89	5.37 ± 0.87	5.48 ± 0.93	<0.001
TG (mmol/L)	0.67 ± 0.32	0.91 ± 0.52	1.55 ± 0.95	2.32 ± 1.02	<0.001
FPG (mmol/L)	4.99 ± 0.33	5.27 ± 0.42	5.48 ± 0.40	5.70 ± 0.26	<0.001
SBP (mmHg)	108.62 ± 10.32	117.38 ± 15.31	125.17 ± 15.71	129.46 ± 15.53	<0.001
DBP (mmHg)	67.69 ± 7.60	73.58 ± 10.59	79.02 ± 10.86	82.07 ± 10.82	<0.001
Drinking status, n (%)					<0.001
Non	4184 (79.35%)	1565 (72.62%)	424 (69.62%)	106 (67.52%)	
Light	587 (11.13%)	287 (13.32%)	83 (13.63%)	21 (13.38%)	
Moderate	366 (6.94%)	216 (10.02%)	70 (11.49%)	20 (12.74%)	
Heavy	136 (2.58%)	87 (4.04%)	32 (5.25%)	10 (6.37%)	
Smoking status, n (%)					<0.001
Never	3401 (64.50%)	1195 (55.45%)	270 (44.33%)	57 (36.31%)	
Past	841 (15.95%)	430 (19.95%)	155 (25.45%)	49 (31.21%)	
Current	1031 (19.55%)	530 (24.59%)	184 (30.21%)	51 (32.48%)	
Years of follow-up	7.19 ± 3.34	7.22 ± 3.29	7.09 ± 3.31	7.24 ± 3.28	0.869
Diagnosis of incident diabetes, n (%)	22 (0.42%)	49 (2.27%)	28 (4.60%)	24 (15.29%)	<0.001

GGT, Gamma-Glutamyl Transferase; BMI, body mass index; ALT, alanine aminotransferase; LDL-C, low-density lipoprotein cholesterol; AST, aspartate aminotransferase; HDL-C, high-density lipoprotein cholesterol; DBP, diastolic pressure; SBP, systolic blood pressure; FPG, fasting plasma glucose; TC, total cholesterol; TG, triglyceride.

### Diabetes prevalence and incidence based on the components of MetS at baseline

**Figure 2:**
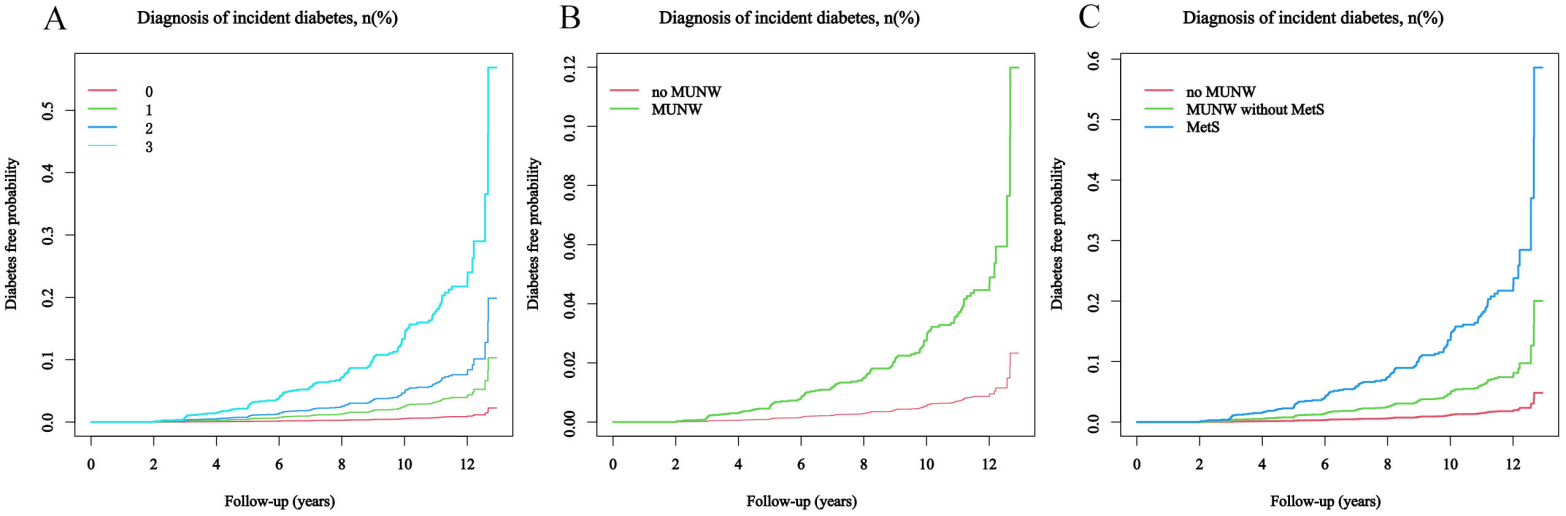
The probability of developing diabetes during the follow-up period, considering the score of MetS components. The patients were stratified into subgroups for analysis: **(A)** by the number of MetS components present: 0, 1, 2, or ≥3; **(B)** by the absence or presence of MUNW; **(C)** into categories of no MUNW, MUNW without MetS, and MetS.

As illustrated in **Figure 2**; **Table 2**, the prevalence, incidence, and associated risk of diabetes escalate in correspondence with the cumulative score of MetS components. Notably, the data revealed that individuals without any MetS components (Group A, 0 components) exhibited the lowest diabetes prevalence at 0.42%, while those with three or more components exhibited the highest prevalence at 15.29%. Similarly, the incidence rate of diabetes showed a marked increase from 0.58 in those with no component to 21.10 in those with at least three MetS components. Compared to these with no MetS component, participants with 1, 2, and ≥3 MetS components had progressively higher adjusted HRs of 4.56, 8.79, and 25.16, respectively. In Group B, the prevalence of diabetes was 0.96% among participants without MUNW, compared to 4.60% in those with MUNW but without MetS, and a striking 15.28% in the MetS group. The corresponding diabetes incidence rates were 1.33, 6.49, and 21.10 per 1000 person-years, respectively. After controlling for confounders, the MUNW group demonstrated an increased risk with an adjusted HR of 5.10, the MUNW without MetS group had an adjusted HR of 3.73, and the MetS group had a notably elevated adjusted HR of 10.12 for developing diabetes.

**Table 2:** Diabetes prevalence and incidence of diabetes on MetS components.

MetS components	Prevalence (%)	Incidence (per 1000 persons peryear)	Adjust model	
			HR (95% CI)	P value
Group A				
0	0.42%	0.58	Ref.	–
1	2.27%	3.15	4.56 (2.73, 7.63)	<0.0001
2	4.60%	6.49	8.79 (4.90, 15.76)	<0.0001
≥3	15.29%	21.10	25.16 (13.32, 47.53)	<0.0001
Group B				
no MUNW	0.96%	1.33	Ref.	–
MUNW without MetS	4.60%	6.49	3.73 (2.37, 5.88)	<0.0001
MetS	15.28%	21.10	10.12 (6.10, 16.81)	<0.0001
Group C				
no MUNW	0.96%	1.33	Ref.	–
MUNW	6.79%	9.53	5.10 (3.48, 7.47)	<0.0001

The prevalence and incidence of diabetes are shown in three groups (Group A, Group B, and Group C). Group A shows statistics for participants with varying numbers of MetS components. Group B classifies participants based on whether they belong to the MUNW category and whether they have MetS. Group C provides a further breakdown of Group B’s data, specifically analyzing MUNW participants and comparing the risk differences with and without MetS.

### The influence of age and gender on the association between components of MetS and diabetes risk

**Figure 3:**
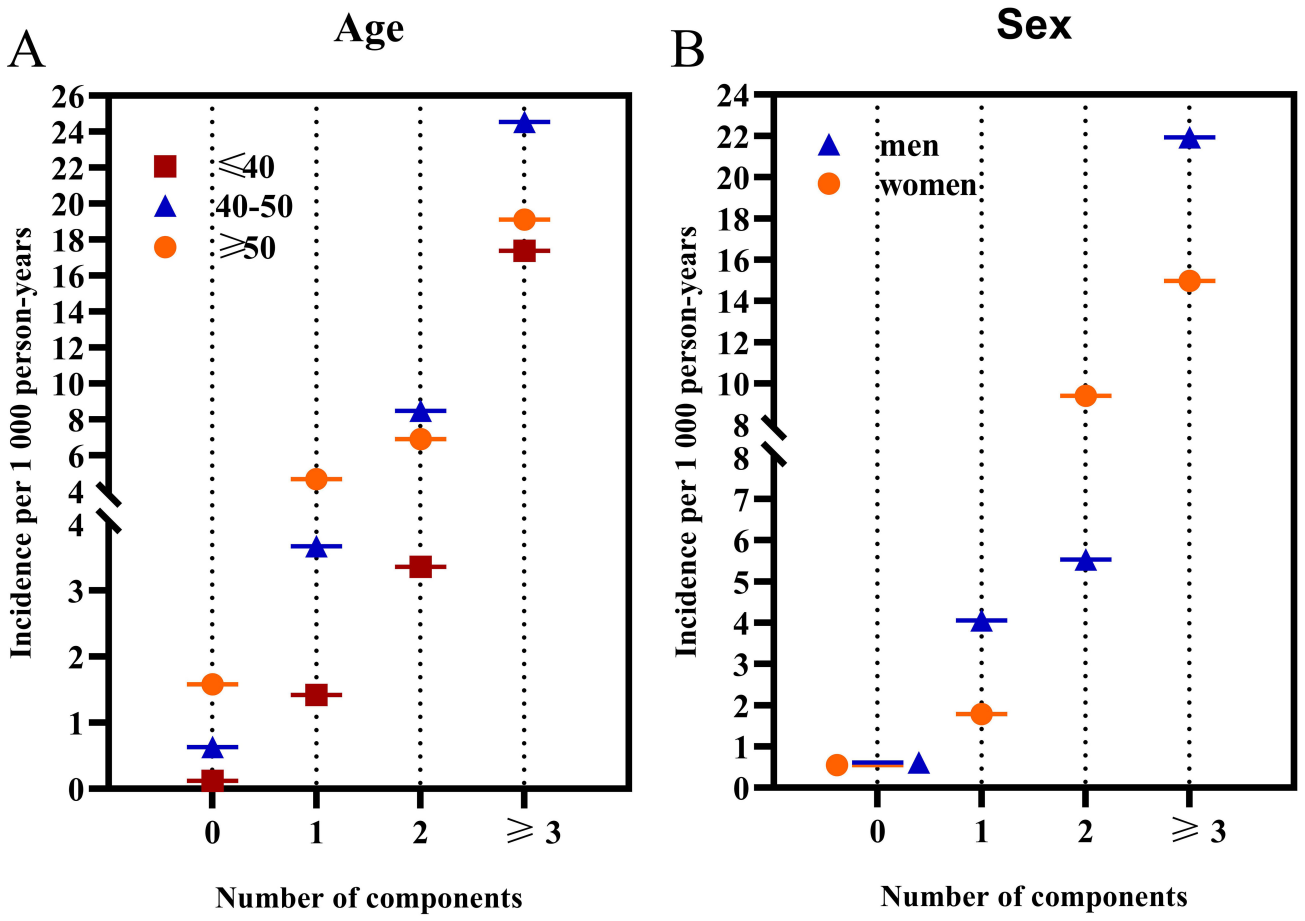
The incidence rates of diabetes categorized by age and gender based on the number of MetS components. **(A)** illustrates the incidence categorized by age; **(B)** displays the incidence categorized by sex.

The incidence of diabetes, stratified by age and gender, notably escalated in parallel with an increasing score of MetS components (**Figure 3**). For participants aged ≥50 years, the incidence rates were 1.58, 4.68, 6.90, and 19.12 for those with 0, 1, 2, and ≥3 MetS components, respectively. For participants aged ≤40 years, the incidence rates were 0.12, 1.42, 3.36, and 17.37 for the corresponding categories; while for those aged 40-49 years, the rates were 0.63, 3.67, 8.48, and 24.55. The HRs for individuals aged ≥50 years with 1, 2, and at least 3 components were 2.92, 4.66, and 12.28, respectively; for those aged 40-49 years, the HRs were 5.50, 11.59, and 33.68; and for those aged ≤40 years, the HRs were 12.31, 25.57, and 129.82, compared to those without any components. These findings underscore the escalating risk of diabetes as the score of MetS components increases, especially in younger age groups.

When stratified by sex, men exhibited higher incidence rates of diabetes compared to women across all age groups. Specifically, for men, the incidence rates were 0.61, 4.05, 5.53, and 21.92 for those with 0, 1, 2, and ≥3 MetS components, respectively. In contrast, for women, the rates were 0.55, 1.78, 9.40, and 14.97 for the same categories. Men presenting 1 and 3 MetS components had a markedly increased diabetes incidence relative to women. However, the gender difference was nonsignificant for individuals with 0 and 2 MetS components (P = 0.40). For men, the HRs for 1, 2, and at least 3 components were 5.61, 7.80, and 28.59, respectively; for women, the HRs were 3.08, 14.65, and 21.16. Finally, a significant interaction was found between MetS components and age, whereas no significant interaction was observed between the MetS components and sex.

## Discussion

This is the first comprehensive longitudinal study to investigate the prevalence of MUNW and its association with diabetes risk among non-diabetic Japanese adults with normal body weight. The findings provide important insights into the metabolic health of this population and highlight the need for targeted prevention strategies, particularly for younger men. The key findings are: 1) The prevalence of MUNW was 9.35%, while 1.92% had MetS. 2) Higher MetS components score was associated with increased diabetes incidence and risk, regardless of demographics. 3) Men aged ≤40 years had a markedly elevated diabetes risk.

Previous studies have shown that the prevalence of the MUNW phenotype ranges from approximately 6.4% to 30% in the global normal-weight population (15, 16). The prevalence of MUNW individuals in Korea can vary widely, ranging from 3.7% to 37.9%, depending on the specific diagnostic criteria used for metabolic health classification (17). American Hispanic populations also fluctuates significantly, ranging from 3.5% to 75% (18). In the Health Improvement Network cohort study, the prevalence of MUNW ranged from 0.4% to 11% (19). However, the prevalence of MUNW varies among lean individuals in the United States, as per the NCEP ATP III criteria, ranging from 4.4% to 12.5% (20). This study utilized the NCEP ATP III criteria and found a MUNW prevalence of 9.35% among normal-weight, non-diabetic Japanese individuals.

Our study revealed that individuals aged ≤40 years had the lowest incidence of diabetes, which aligns with findings from comparable research (21). However, stratified analysis results show that the risk of diabetes is highest in individuals aged ≤40 years. Since the 2000s, the greatest relative increases in both incidence and prevalence of type 2 diabetes have been observed among younger adults under the age of 40, as well as in adolescents and even children (22). Patients with young-onset type 2 diabetes, typically those under the age of 40, often exhibit more severe insulin resistance and a more rapid decline in β-cell function (23). This suggests that early-onset diabetes may present a more aggressive disease phenotype. Genetic factors and exposure to hyperglycemia during pregnancy may be important risk factors contributing to young-onset disease (24). Despite shorter diabetes duration, younger people with diabetes exhibited poorer diabetes self-care practices, including greater difficulty following dietary recommendations, more frequent medication forgetfulness, less frequent blood glucose monitoring, and poorer glycemic control, compared to older people (25). This group would likely benefit from customized support to promote lifestyle modifications and enhance their diabetes care (26). This finding underscores the significant rise in diabetes prevalence among U.S. individuals under the age of 20, observed from 2002 to 2018 (27). Additionally, our study revealed that a higher BMI, even within the normal range, was associated with a higher proportion of individuals classified as MUNW. Within the non-obese BMI range, BMI acts as a dose-dependent factor contributing to the risk of diabetes among middle-aged individuals in Japan (28). The most influential factor in predicting adult MUNW was the rise in BMI from childhood to adulthood (29). This indicates a potential association between specific lifestyle factors and the emergence of the MUNW phenotype in adults. Therefore, the increasing prevalence of MUNW in children and young individuals will result in more individuals at risk of developing diabetes at an earlier age. Previous studies have also shown that in Japan, individuals who successfully achieved a 10% or greater reduction in BMI within one year had a diabetes remission incidence rate of 48.2 per 1000 person-years (30). However, for MUNW individuals who already have a normal BMI, further lowering their BMI may not be an effective strategy.

In addition to BMI, particularly, we observed that Japanese men seem to be more susceptible to diabetes. Compared to Europeans, East Asians are more susceptible to diabetes at the same BMI level (31). Therefore, it is advisable to recommend early implementation of dietary and lifestyle interventions tailored to the specific needs of MUNW individuals. previous studies have confirmed that age, family history of previous studies have confirmed that age, family history of, smoking, and BMI are significant risk factors for the MUNW phenotype in men, while alcohol consumption and hypertension are notable risk factors for women (32–34). Diet is one of the most important modifiable risk factors for developing diabetes. Compared to a Prudent dietary pattern, adherence to a Western dietary pattern characterized by high consumption of red and processed meats, alcoholic beverages, refined grains, and sugar-sweetened beverages was associated with an increased risk of developing prediabetes, undiagnosed diabetes, and prevalent diabetes (35). Walnuts, black mulberries, and olives are rich in antioxidant properties and may offer significant benefits for diabetes management and cardiovascular health (36). Japanese individuals prefer sweet flavors; however, a higher dietary glycemic load is correlated with an increased diabetes risk (37, 38). Similarly, rice consumption is associated with a heightened diabetes risk, whereas men who consume more fish exhibit a reduced diabetes risk (39, 40). Therefore, adjusting dietary structure, such as reducing red meat consumption and increasing vegetable consumption, may reduce the risk of metabolic abnormality (41). In addition to dietary pattern, engaging in regular physical activity and adopting strategies to decrease sedentary time, such as taking periodic activity breaks, can positively influence MetS (42). Among diabetes patients under the age of 65, 31.0% do not have a habit of exercising (43). Japanese men with type 2 diabetes who actively engage in leisure-time physical activity tend to experience improved glycemic control, reduced cardiovascular risk factors, a decreased need for insulin, and potential mitigation of retinal aging (44–46).

The incidence and prevalence of diabetes in young individuals (aged under 40 years) have significantly increased in recent years, with rates approximately two to three times higher than before (47). Compared with diabetes of usual onset, patients diagnosed at a younger age are more likely to develop severe microvascular and macrovascular complications and have a significantly shorter life expectancy (48). This emphasizes the need for more vigorous metabolic control in early-onset diabetes.

Given that the BMI of the population in this study is within the normal range, focusing solely on weight control may not be the most effective strategy. Epidemiological studies among non-obese populations have demonstrated that specific dietary qualities and physical activity patterns can impact metabolic risk factors independently of body weight changes (49, 50).However, further research is needed to determine whether lifestyle interventions involving diet and exercise can resolve metabolic dysfunction and facilitate the transition from a metabolically unhealthy to a metabolically healthy phenotype.

### Limitations

This study has several limitations: 1) The exclusion of participants with higher glucose levels and obesity may have underestimated the impact of obesity. 2) The sample was limited to non-diabetic Japanese individuals, so the findings may not be generalizable to other populations. 3) The longitudinal design of the study could be influenced by changing lifestyle and treatment factors. 4) Uncontrolled confounding factors related to sample characteristics may have affected the results.

### Conclusion

This study found that the prevalence of MUNW was 9.35% among non-diabetic Japanese adults. Diabetes risk increased in parallel with the score of MetS components, with young men aged ≤40 years exhibiting the highest risk. Early interventions targeting this high-risk group, particularly young men, are crucial for effective prevention of diabetes.

## Data Availability

The datasets presented in this study can be found in online repositories. The names of the repository/repositories and accession number(s) can be found below: https://datadryad.org/stash/dataset/doi:10.5061/dryad.8q0p192.
